# Developing novel liquid biopsy by selective capture of viral RNA on magnetic beads to detect COVID-19

**DOI:** 10.22038/IJBMS.2022.65260.14379

**Published:** 2022-06

**Authors:** Mohammad Amin Kerachian, Saeid Amel Jamehdar, Marjan Azghandi, Nasrin Keyvanlou, Sina Mozaffari-Jovin, Ali Javadmanesh, Mahnaz Amini

**Affiliations:** 1 Medical Genetics Research Center, Mashhad University of Medical Sciences, Mashhad, Iran; 2 Department of Medical Genetics, Faculty of Medicine, Mashhad University of Medical Sciences, Mashhad, Iran; 3 Genetics Research Unit, Reza Radiotherapy and Oncology Center, Mashhad, Iran; 4 Antimicrobial Resistance Research Center, Mashhad University of Medical Sciences, Mashhad, Iran; 5 Department of Animal Science, Faculty of Agriculture, Ferdowsi University of Mashhad, Mashhad, Iran; 6 Department of Medical Genetics, Shahid Sadoughi University of Medical Sciences, Yazd, Iran; 7 Stem Cell Biology and Regenerative Medicine Research Group, Research Institute of Biotechnology, Ferdowsi University of Mashhad, Mashhad, Iran; 8 Lung Diseases Research Center, Mashhad University of Medical Sciences, Mashhad, Iran

**Keywords:** Bead, Blood, Covid-19, Extraction, RNA, Serum

## Abstract

**Objective(s):**

Early, specific, and sensitive detection methods of COVID-19 are essential for force stopping its worldwide infection. Although CT images of the lung and/or viral RNA extraction followed by real-time reverse-transcriptase-polymerase chain reaction (rRT-PCR) are widely used; they have some limitations. Here, we developed a highly sensitive magnetic bead-based viral RNA extraction assay followed by rRT-PCR.

**Materials and Methods:**

Case group included oropharyngeal/nasopharyngeal and blood samples from 30 patients diagnosed positive by PCR test for COVID-19 and control group included 30 same samples from COVID-19 negative PCR test individuals. RNA was extracted, using viral RNA extraction kit as well as using our hand-made capture bead-based technique. A one-step cDNA synthesis and Real Time PCR was conducted. A two-step comparison of the different viral RNA extraction methods for oropharyngeal/nasopharyngeal and blood samples was performed. Student t-test was applied with a *P<*0.05 considered statistically significant.

**Results:**

In the case group, all 30 mucosal samples extracted either with viral RNA extraction kit or with beads-based assay were COVID-19 positive although in the latter category, Cqs were much lower. Although 43% of plasma samples extracted by bead-based method were found to be positive but no plasma samples extracted with column-based kit were detected positive by Real Time PCR.

**Conclusion:**

Bead-based RNA extraction method can reduce RNA loss by its single-tube performance and enhance the test sensitivity. It is also more sensitive to lower viral loads as shown in the detection of blood samples and the lower Cqs of mucosal samples.

## Introduction

The Severe Acute Respiratory Syndrome Coronavirus 2 (SARS-CoV-2) is a new virus that emerged in China in December 2019 and belongs to the betacoronavirus family (1-3). This virus is the cause of the coronavirus disease 2019 (COVID-19), which the World Health Organization (WHO) declared a Public Health Emergency of International Concern (PHEIC) on January 30, 2020, and a pandemic on March 11, 2020 (4). 

The rapidly increasing number of COVID-19 infections and deaths has defined SARS-CoV-2 as a global threat to public health and has imposed numerous burdens on the healthcare system, including the availability of diagnostic tests (5-7). Clinical diagnosis is difficult because of the various clinical manifestations of SARS-CoV-2 infection, which can range from no or mild acute respiratory disease to severe pneumonia and acute respiratory distress syndrome (8, 9). Therefore, an accurate laboratory method is essential for confirming a diagnosis of COVID-19 (10).

The nucleic acid test serves as the gold standard method for the etiological diagnosis of SARS-CoV-2 infection. However, the large demand for real-time reverse-transcriptase-polymerase chain reaction (rRT-PCR) tests due to the worldwide extension of the virus is highlighting the limitations of this type of diagnosis on a large scale. These include the long turnaround times (on average over 2-3 hr to generate results) and the need of certified laboratories, expensive equipment, and trained personnel (11). Several diagnostic tools for SARS-CoV-2 infections have been developed since January 2020. Although these tools can help to detect SARS-CoV-2, various limitations have been identified during their clinical application (12). Furthermore, an increasing number of commercial and in-house diagnostic tests has been developed, although most have not yet obtained FDA approval (12). However, diagnostic capacity has yet to meet current demands in many areas, and widespread tests or test-seeking is likely to overwhelm healthcare systems. Overall, balancing the increasing use of laboratory-developed tests, the risk of test errors, the need for tests, the burden on healthcare systems, the benefits of early diagnosis, and the risk of unnecessary exposure remain as the significant challenges.

According to WHO, while respiratory samples have the highest yield, other samples, including stool and blood, can detect coronavirus. However, owing to the low sensitivity of the blood test, it is not widely used at present (13-15).

It has been shown that viral RNA in plasma or serum could be detected in COVID-19 patients on the first 2 or 3 days after onset of symptoms; but there are no data on the viral load in plasma and serum, among individuals in the incubation period (16, 17). In the first forty-one patients in the city of Wuhan, viremia was found in 6 out of 41 (15%) patients. The median PCR cycle threshold value was 35.1 (95% CI: 34.7-35.1), suggesting a very low RNA concentration with no difference found between intensive care unit patients and patients with mild symptoms (18). It should be declared that sometimes the virus can be detected in blood and rectal swabs but not found in the throat samples, with the result that these patients can act as carriers and transmit the infection to other people, which shows the importance of testing samples from different sources to confirm the infection (19, 20). 

The primary challenge for identification of viral RNAs is their minute amounts in samples. Besides, large amounts of background RNA can mask the low abundance of viral RNA fragments, leading to false negative results (21). Thus, maximizing target viral RNA recovery is critical to enhance the sensitivity. Recent studies have highlighted the absence of a standard method in RNA extraction, and have compared various extraction techniques demonstrating the importance of nucleic acid quantity isolated for the outcomes of detection assays (22). To maximize the sensitivity of diagnostic tests in patients suffering from COVID-19, increasing the amount and purity of extracted RNA along with an optimal extraction method are key players to reduce false-negative results. The aim of this study is to establish and optimize a novel and sensitive approach for solving technical problems of the new coronavirus (SARS-CoV-2) RNA detection in nasopharyngeal or oropharyngeal swabs as well as in plasma specimens by a magnetic bead-based capture approach.

## Materials and Methods

***Sample collection***

All patients gave written informed permission to retain and analyze their samples for this study. All procedures and protocols in the present study were approved by the ethical committee of the National Institute for Medical Research Development (NIMAD) with the national ethical # IR.NIMAD.REC.1399.090. Following informed approval, a minimum of 30 oropharyngeal/nasopharyngeal samples (positive for the routine real-time RT-PCR diagnostic test) and 30 blood samples (2 ml each) from the same patients, as well as 30 oropharyngeal/nasopharyngeal samples from normal individuals as control (negative for the routine real-time RT-PCR diagnostic test) and their blood samples (2 ml each) were recruited for our pilot study. The case group was selected from patients with clinically positive symptoms and signs for COVID-19 (with positive RT-PCR tests and X-ray images) recruited from the COVID referral hospitals. The clinical characteristics of the patients with covid-19 disease is listed in Table 1.

***Isolation phase (viral RNA extraction)***

**Figure 1:**
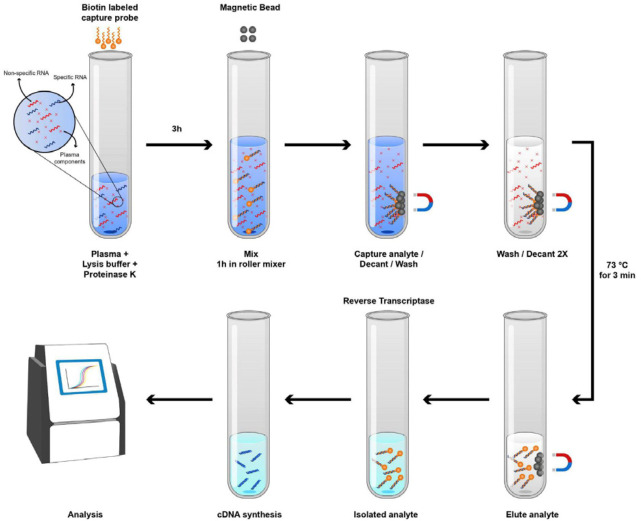
Selective extraction of viral RNA workflow

The extraction of viral RNA from oropharyngeal/nasopharyngeal and blood samples was performed by a sensitive capture bead-based technique illustrated in Figure 1. 

*Streptavidin capture bead-based RNA extraction*

For the capture bead-based technique, coronavirus RNA fragments were pulled down from the total nucleic acid preparations by performing oligonucleotide hybrid captures. Biotinylated oligonucleotides targeting genes encoding E and N genes as well as beta-actin (ACTB) RNA fragments (internal control) were designed and synthetized. To extract viral RNA, 200 µl of oropharyngeal/nasopharyngeal samples or plasma obtained from each patient as well as normal individuals were mixed with an equal volume of the lysis buffer (60 mM Tris-HCl, pH 7.4, 30 mM Na2EDTA, 6 M Guanidium-HCl, 10 % Tween 20 and 2% Triton X-100). Then, 20 µl of 20 mg/ml proteinase K was added and the mixture was incubated for 30 min at 56 ^°^C. The capture assay is conducted by adding 420 µl of the mixture of plasma and the lysis buffer to an equal volume of 6M guanidine isothiocyanate (GITC) solution containing a biotinylated sequence-specific capture probe at a concentration of 20 pmol. Then the mixture was incubated for 3 hours at room temperature (RT) on a roller mixer with a gentle speed. Five microliters of streptavidin-coated magnetic beads (Dynabeads™ M-280 Streptavidin, Invitrogen) were added to the solution, and the tubes were incubated for an additional 3 hours at RT on the roller mixer (same speed). The bead/capture probe complexes were then washed twice with washing buffer, and the sequence-specific captured RNA was eluted into 10 µl ddH2O by heat denaturation (72 ^°^C for 3 min) and was used as a template for real-time RT-PCR.

In parallel to RNA extraction from patient and control oropharyngeal/nasopharyngeal and blood samples by our method (Figure 1), RNA extraction was performed using a standard commercial viral RNA extraction kit (Viragene, Iran) on the same samples using 200 µl of oropharyngeal/nasopharyngeal samples or plasma. The latter extracted RNA samples (10 µl) were used in a comparative analysis to evaluate the sensitivity and reproducibility of our capture bead-based technique.

***Detection phase***

In this study, according to the Centers for Disease Control and Prevention (CDC) and WHO guidance, TaqMan-based-diagnostic RT-PCR assays were conducted using E and N as well as ACTB genes-specific primers for detection of COVID-19. 

At the detection phase, the results from the streptavidin capture bead technique and the commercial viral RNA extraction kit approaches were compared for both oropharyngeal/nasopharyngeal and blood specimens. In addition, the results from oropharyngeal/nasopharyngeal swabs were compared with that of the blood samples in each approach. 

***Statistical analysis***

Selectivity was determined using the Student t-test by comparing the Cq values obtained from the two viral RNA extraction approaches. Probability values (*P-values*) was determined from the t-test results, and a significance level of 0.05 was chosen. 

## Results

After setting up and optimizing the capture bead method, a pilot study was conducted on Covid-19 patients. Briefly, 30 oropharyngeal/nasopharyngeal samples (positive for the routine RT-PCR diagnostic test), 30 blood samples from the same patients and 30 oropharyngeal/nasopharyngeal samples from normal individuals’ matching the sex and age as control (negative for the routine RT-PCR diagnostic test) was entered to the pilot study. The case group was selected from patients with clinically positive symptoms and signs for Covid-19 (with positive RT-PCR tests and X-ray images). The clinical characteristics of patients were shown in table 1. Many patients (96.6%) had an underlying disease. The pilot study results showed that all 30 mucosal samples, either extracted with column-based kit or extracted using magnetic bead, were found to be positive. The comparison of the Cq values of column-based and magnetic bead-based extraction kits in mucosal and plasma were demonstrated in Table 2. 

**Table 1:** Clinical characteristics of patients with Covid-19 disease

Age (year)	61.33
**Gender**	
Men	18(**60%**)
Women	12 (**40%**)
**Clinical Characteristics**	
Asymptomatic	0 (**0%**)
Symptomatic	30(**100%**)
Mild: cough, fever, sore throat	4(1**3.33%**)
Moderate: fever, deep cough, fatigue, body aches	7 (**23.33%**)
Severe: Shortness of breath, diarrhea, chest pain	17 (**56.66%**)
**Underlying diseases**	
Diabetes	9 (**30%**)
Hypertension	9 (**30%**)
Pulmonary disease	7 (**23.33%**)
Hepatic disease	2 (**6.66%**)
Heart disease	13 (**43.33%**)
Cerebral disease	10 (**33.33%**)
Thyroid disease	0 (**0%**)
Malignancy	9 (**30%**)
**Co-infection**	
Fungi	1 (**3.33%**)
Bacteria	4 (**13.33%**)

The extraction of viral RNA from oropharyngeal/nasopharyngeal and blood samples was performed by a sensitive capture bead-based technique illustrated here. Biotin labeled capture probes were added to the plasma with lysis buffer and proteinase K. Following the capture of viral RNA and sets of washing steps, reverse transcription was performed, and the cDNA was transferred to the real time PCR for amplification

**Table 2:** The comparison of the Cq values of column-based and magnetic bead-based extraction kits in mucosal and plasma

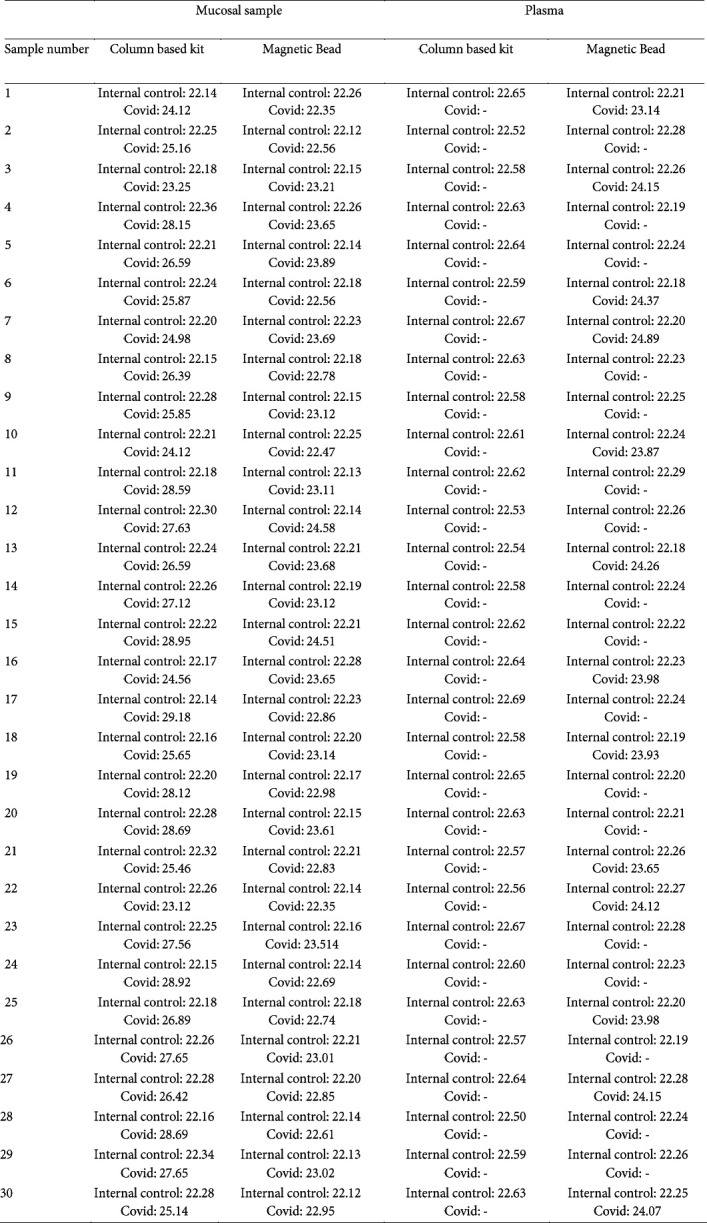

## Discussion

The average age of patients with Covid-19 was about 61 years with 60% male and 40 % female, mostly clinically categorized in the severe form of the disease, suffering from shortness of breath, diarrhea and/or chest pain (Table 1). Many patients had an underlying disease with a predominance of heart and cerebral diseases. As shown in Table 2, the Cq values obtained from the samples extracted using magnetic beads were lower than those obtained from the samples used in the column-based kits, indicating a higher quantity and quality of RNAs extracted using magnetic beads. In the case of plasma samples, as shown in Table 2, none of the samples extracted using the column-based kit were detectable by Real time PCR, which may be due to the low virus load in the blood samples and the inability of the column-based kits to extract and purify the low levels of RNA. Furthermore, the high-performance PCR reaction may not be possible due to the presence of nonspecific RNAs and the low viral RNA load in the extracted sample.

According to the results of this study, the magnetic bead-based selective extraction method has a higher quality than conventional column-based methods and has a higher performance in cases where the load of the target virus is low in the blood. Although the sensitivity relied on the capture bead-based technique is not very high (43%), in cases which the patient is in coma and oropharyngeal/nasopharyngeal is contraindicated, blood sampling followed by magnetic selective extraction could be an alternative approach. Furthermore, this novel technology could be used not only for coronavirus but also for any mutated coronavirus or even other future attacking viruses with some modifications. Since there is no single tube isolation/detection kit for Covid-19 in the marketplace, this method has the potency to revolutionize the diagnosis of the virus.

In previous studies, viral RNA extraction has been already performed using magnetic beads (23-28). However, the differences between the current study and previous ones include a) previously silica-based magnetic beads were used, while in this study, we used streptavidin-coated magnetic beads. Silica-based magnetic beads extract the total RNA in compared to the selective viral RNA method which reduces the impurity and leads to extremely sensitive detection. It is feasible that the high protein levels in plasma could obscure the effect of the scanty viral RNA using the silica-based methods. b) To the best of our knowledge, this study is the only study that has used selective RNA extraction to detect Corona virus on infected blood samples. Recently, a study demonstrated using magnetic capture probes with HRP-labeled reporters in SARS-CoV-2 RNA extraction limited to nasal swabs (29). 

Furthermore, the capture bead-based technique has the possibility to perform RNA extraction, cDNA synthesis and even the detection in a single-tube, thereby minimizing the RNA loss at each step and thus improving the sensitivity of the test. Minimization of wash/binding steps would diminish RNA loss at each step, and sample processing in a single tube could reduce RNA loss during tube transfers. This feature allows us to set up the optimized assay in a 96-well plate format, which will significantly enhance the patient screening capacity. Moreover, this single-tube approach has an enormous potential for development to a fully automated platform for diagnosis of COVID-19 in clinical laboratories.

## Conclusion

To the best of our knowledge, this is the first study using capture beads to isolate viral RNA of coronavirus from fluid bodies such as blood. Since we expect an improvement in the pre-analytic phase, the number of false negatives in the coronavirus detection will remarkably drop in compared to the column-based extraction methods. 

## Funding

The authors received funding for this work from the National Institute for Medical Research Development (NIMAD), Project No. 993860 and Mashhad University of Medical Sciences, Project No. 981856.

## Authors’ Contributions

MAK, SAJ, MA, NK, SMJ, AJ, MA participated in study design, data analysis, and preparation of the drafted manuscript. MAK, MA, AJ and MA revised the manuscript. All authors read and approved the final manuscript.

## Conflicts of Interest

The authors declare that they have no conflicts of interest.
